# The Thrombin Receptor Restricts Subventricular Zone Neural Stem Cell Expansion and Differentiation

**DOI:** 10.1038/s41598-018-27613-9

**Published:** 2018-06-19

**Authors:** Chan-Il Choi, Hyesook Yoon, Kristen L. Drucker, Monica R. Langley, Laurel Kleppe, Isobel A. Scarisbrick

**Affiliations:** 1 Department of Physical Medicine and Rehabilitation Mayo Clinic, Rochester, MN 55905 USA; 2 Rehabilitation Medicine Research Center Mayo Clinic, Rochester, MN 55905 USA; 3 Department of Physiology Mayo Clinic, Rochester, MN 55905 USA

## Abstract

Thrombin is frequently increased in the CNS after injury yet little is known regarding its effects on neural stem cells. Here we show that the subventricular zone (SVZ) of adult mice lacking the high affinity receptor for thrombin, proteinase activated receptor 1 (PAR1), show increased numbers of Sox2+ and Ki-67+ self-renewing neural stem cells (NSCs) and Olig2+ oligodendrocyte progenitors. SVZ NSCs derived from PAR1-knockout mice, or treated with a PAR1 small molecule inhibitor (SCH79797), exhibited enhanced capacity for self-renewal *in vitro*, including increases in neurosphere formation and BrdU incorporation. PAR1-knockout SVZ monolayer cultures contained more Nestin, NG2+ and Olig2+ cells indicative of enhancements in expansion and differentiation towards the oligodendrocyte lineage. Cultures of NSCs lacking PAR1 also expressed higher levels of myelin basic protein, proteolipid protein and glial fibrillary acidic protein upon differentiation. Complementing these findings, the corpus callosum and anterior commissure of adult PAR1-knockout mice contained greater numbers of Olig2+ progenitors and CC1+ mature oligodendrocytes. Together these findings highlight PAR1 inhibition as a means to expand adult SVZ NSCs and to promote an increased number of mature myelinating oligodendrocytes *in vivo* that may be of particular benefit in the context of neural injury where PAR1 agonists such as thrombin are deregulated.

## Introduction

The subventricular zone (SVZ) of the forebrain lateral ventricles and the subgranular zone (SGZ) in the hippocampal dentate gyrus are neurogenic niches in the adult brain that contain multipotent cells that self-renew and differentiate into all neural cell types including neurons, astrocytes and oligodendrocytes. NSCs are generated in these regions of the adult murine brain throughout life and play a critical role in replacement of post mitotic cells and therefore in regenerative repair after injury^1–4^. Although there is now considerable evidence regarding the differentiation and migration patterns of NSCs from these regenerative niches, far less is known regarding environmental factors present that may regulate their expansion and differentiation in the intact and injured CNS.

Emerging studies show that a family of G-protein coupled receptors (GPCRs) referred to as proteinase activated receptors (PARs) are densely expressed in the adult CNS and can regulate the proliferation and differentiation of both neurons and neuroglial cells. While all four family members (PAR1-4) are expressed at significant levels across the brain and spinal cord^5,6^, PAR1 is by far the most abundant^6,7^. PARs are activated by cleavage within their extracellular domain revealing a new amino-terminus that binds to the receptor’s second extracellular loop thereby eliciting intracellular signaling. PARs are of particular interest clinically as their activating proteinases are widely deregulated in the context of CNS injury and disease^8^. For example thrombin, which readily extravasates with CNS injury, is the high affinity agonist for PAR1. PAR1 may also be activated by matrix metalloproteinases 1 (MMP1)^9^, certain kallikreins^6,7,10–12^, tissue plasminogen activator and plasmin^13^. PAR1 is therefore positioned to translate changes in the proteolytic microenvironment into changes in cell behavior. For example, PAR1 activation promotes proliferation of adult brain astrocytes^14^. PAR1 activation also suppresses differentiation of oligodendrocyte progenitor cells, while a PAR1 small molecule inhibitor enhances differentiation and production of myelin related genes^15,16^. Indeed, mice with PAR1 knockout show an accelerated pattern of myelin production in the spinal cord developmentally and higher levels of myelin basic protein (MBP) and thicker myelin sheaths in adulthood^16^. Notably, recent studies show that proliferation of NSCs derived from the SGZ of the hippocampus are inhibited by thrombin or a PAR1 activating peptide (PAR1-AP) and blockade of serine proteinases by intracerebroventricular infusion of a pan-serine proteinase inhibitor AEBMSF increased SGZ cell proliferation, although the cell type involved was not investigated^17^. PAR1 is also documented to play a role in cell proliferation, differentiation, and migration in other adult stem/progenitor cell niches, including endothelial progenitor cells^18,19^ and hematopoietic stem cells^20,21^. Since orally bioavailable PAR1 small molecule inhibitors are already available in the clinic^22–25^, a better understanding of the physiological activities of PAR1 towards neural stems of the adult brain may point to new therapeutic avenues to enhance the generation of new neurons and neuroglia.

Given accumulating evidence that PAR1 is positioned to regulate neural stem/progenitor cell development and its critical activities in the production of myelinating cells^16^, we investigated the role of PAR1 in the production of neural progenitor cells arising from the SVZ of the adult mouse brain and their differentiation towards a mature myelinating phenotype. Using NSCs derived from the SVZ of adult wild type or PAR1 knockout mice, our results show for the first time that genetic PAR1 loss-of-function promotes expansion of SVZ neural stem cells *in vitro* and *in vivo*. Moreover, our findings suggest that NSCs lacking PAR1 show enhancements in differentiation towards the oligodendrocyte lineage *in vitro*, an effect that may account for the higher numbers of oligodendrocytes also observed in CNS white matter tracts in mice lacking PAR1. Together these findings highlight PAR1 as a new target for inhibition in therapeutic strategies aimed at fostering NSC expansion and the production of oligodendrocyte progenitors (OPCs) and mature myelinating oligodendroglia that may be useful for restoration of function in the context of injury and diseases of the adult CNS.

## Materials and Methods

### Animal Care and Use

PAR1-null mice (PAR1−/−, B6.129S4-F2r^tm1Ajc^/J) were obtained from Jackson Labs (Bar Harbor, ME) and have been crossed with C57BL6/J for more than 40 generations^16,26^. Age matched PAR1+/+ served as controls. All studies used a combination of male and female mice, except for histological analyses that used on male mice only. The Mayo Clinic Institutional Animal Care and Use Committee approved all surgical procedures and experimental manipulations. All experiments were conducted in accordance with the institutional guidelines and regulations for animal experiments.

### Adult Mouse Neural Stem Cell Isolation and Culture

Neural stem cells (NSCs) derived from the subventricular zone (SVZ) were isolated as described previously^27^. Briefly, 8-week-old PAR1+/+ or PAR1−/− mice were deeply anesthetized by an overdose of pentobarbital and decapitated. The brains were removed from the skull and were placed in cold Dulbecco’s phosphate buffered saline (DPBS), cut into 1 mm thick coronal sections, and the SVZ of each hemisphere was dissected. Neural stem cells were dissociated from the SVZ using Accutase (1X), and gown in Dulbecco’s modified Eagle Medium/F12 with B27 supplement (1X), Antibiotic-Antimycotic (1X), insulin (20 µg/mL), epidermal growth factor (EGF, 20 ng/mL) and basic fibroblast growth factor (bFGF, 20 ng/mL, PeproTech, Rocky Hill, NJ). All cell culture products were obtained from Life Technologies (Carlsbad, CA), unless otherwise stated. Neural stem cells were grown and passaged in suspension as neurospheres in tissue culture treated flasks.

### Expression of functional PAR1 by SVZ neural stem cells

#### PAR1 immunostaining and *in situ* hybridization

The association of PAR1 with NSCs located in the SVZ of the adult (8 wk) PAR1+/+ mouse brain was determined in 4% paraformaldehyde fixed paraffin embedded 6 μm sections using immunofluorescence and *in situ* hybridization techniques. PAR1 immunoreactivity was detected with a monoclonal antibody (NBP1-71770, Novus Biologicals, Littleton, CO). PAR1 was co-localized with either Nestin (NB100-1604, Novus Biologicals, Littleton, CO), or Sex determining region Y-box 2 (Sox2, ab97959, Abcam, Cambridge, MA). All species appropriate fluorochrome conjugated secondary antibodies were obtained from Jackson ImmunoResearch (West Grove, PA). Sections were cover slipped with Vectashield HardSet containing 4′,6-diamidino-2-phenylindole (DAPI, Vector Laboratories, Burlingame, CA). Slides were imaged on the LSM 780 inverted confocal microscope (Carl Zeiss, Inc., Thornwood, NY).

To confirm the expression of PAR1 by Sox2 and Nestin positive cells in the SVZ of the adult mouse brain, sections parallel to those for immunohistochemistry were examined for RNA expression by *in situ* hybridization using RNAscope 2.5 HD Duplex reagents (#322430, Advanced Cell Diagnostics, Newark, CA). Probes specific for PAR1 (Mm-F2R-C1, 471081), Sox2 (Mm-Sox2-C2, 401041) and Nestin (Mm-Nes-C2, 313161) were hybridized as previously detailed^28^. The C1-tagged PAR1 probe was visualized using horseradish peroxidase-based green chromogenic development and the C2-tagged Sox2 or Nestin probes using alkaline phosphatase-based fast red color development. Sections were counterstained with hematoxylin and cover slipped with Vectamount (H5000, Vector Labs, Burlingame, CA).

#### PAR1 RNA expression in cultured NSCs

The potential for PAR1 to exert a regulatory role in neural stem cell expansion and/or differentiation was examined by quantifying PAR1 or Nestin (a marker of stem cell status) RNA expression in Passage 3 neurospheres, or in dissociated NSCs plated on poly-L-lysine coated (10 μg/ml) 6 well plates for 2 days *in vitro* (DIV) in the presence of EGF and bFGF, or after withdrawal of these growth factors for 5 DIV to induce differentiation. Total RNA was harvested using RNA Stat-60 (Tel-Test, Inc., Friendswood, TX), and RNA expression levels were determined in 0.125 μg of RNA using quantitative real-time reverse-transcription polymerase chain reaction (RT-PCR, CFX96 Touch Real-Time RCR Detection System, Bio-Rad Laboratories, Hercules, CA). Primers specific for *Mus musculus* PAR1 (NM_010169) were forward, 5′-CAGCCAGAATCAGAGAGGACAGA-3′ and reverse, 5′-GGAAGGCTGACAATGAACACAATC-3′ and were obtained from Integrated DNA Technologies (IDT, Skokie, IL). Primers for *Mus musculus* Nestin (NM_016701, ID: Mm00450205_m1) were from Applied Biosystems (AB, Foster City, CA). To control for loading the housekeeping genes Rn18s (AB ID: Mm03928990_g1) and glyceraldehyde-3-phosphate dehydrogenase (GAPDH, AB ID: Mm99999915_g1) were amplified in the same RNA samples.

#### Ca^2+^ Imaging

The ability of SVZ neural stem cell PAR1 to gate Ca^2+^ signaling was determined using a series of receptor cross desensitization experiments involving the canonical PAR1 activating enzyme, thrombin, as well as a peptide that specifically activates PAR1 (PAR1-AP). Dissociated neural stem cells were plated at a density of 21,400 cells/cm^2^ on Poly-L-lysine coated (10 μg/mL) glass chambered slides (Cat #155409, Nalge Nunc International, Roskilde, Denmark). Prior to Ca^2+^ imaging, cells were loaded with the Rhod-3 calcium indicator (Cat #R10145, Life Technologies). In all experiments, changes in Rhod-3 fluorescence intensity after application of PAR1 agonists were captured with a 20X objective on the LSM 780. A receptor cross desensitization strategy was used to determine the extent to which changes in fluorescence observed after application of the first agonist were due to PAR1 activation. In this case, Rhod-3 loaded NSCs were imaged for 2 min to establish the baseline fluorescence prior to application of either thrombin (5 μg/ml (135 nM), HT1002a, Enzyme Research Laboratories, South Bend IN), or PAR1-activating peptide (PAR1-AP, TFLLR-amide, 40 μM, Peptides International, Louisville, KY), and the alternative agonist was applied 4 min after application of the first agonist with changes in fluorescence monitored for an additional 4 min. The disruption of PAR1-mediated calcium signaling in the NSCs lacking PAR1 was confirmed using NSCs derived from PAR1−/− mice. Rhod-3 loaded NSCs were imaged for 1.5 min to establish the baseline fluorescence prior to application of either thrombin, or PAR1-AP, with changes in fluorescence monitored over the subsequent 3.5 min. Cells were selected across multiple; wells for analysis, with a minimum of 15 cells, and all results were verified using independent cell culture preparations. A baseline intensity value (F_0_) was created for each cell using the minimum intensity value collected. The intensity (F) of fluorescence emission responses were expressed as the mean ΔF/F_0_ = [(F − F_0_)/F_0_] and a line graph was created to illustrate data collected at one second intervals over the entire period of analysis.

### Regulatory role of PAR1 on NSC proliferation *in vitro*

#### Neurosphere formation assay

To address whether changes in PAR1 activation affect the proliferation of neural stem cells *in vitro*, suspensions of passage 3 PAR1+/+ or PAR1−/− neurospheres were enzymatically dissociated with Accutase and passed through a 40 μm filter to ensure a single cell suspension. Cells were plated and grown in triplicate at low density (5000 cells/well) in 6 well plates. After 7 days, the total number of neurospheres per well was quantified.

#### BrdU incorporation assay

To confirm the potential for PAR1 activation or inactivation to regulate neural stem cell proliferation, we next performed a bromodeoxyuridine (BrdU) incorporation assay (2750, EMD Millipore, Billerica, MA). In each case, dissociated NSCs were plated at a density of 25,000 cells in 96 well tissue culture plates for 24 h, before the addition of PAR1 antagonists or agonists, in addition to BrdU (1X), for an additional 18 h culture period. The impact of PAR1 inactivation was determined by comparison of BrdU incorporation by PAR1+/+ or PAR1−/− NSCs. Alternatively, the effect of PAR1 inactivation was evaluated by application of a PAR1 specific small molecule inhibitor (SCH79797, 35 or 70 nM, Tocris, Bristol, United Kingdom) applied to PAR1+/+ NSCs. Conversely, the impact of PAR1 activation was evaluated by application PAR1-AP (40 μM) to PAR1+/+ NSCs. Vehicle (DPBS) alone, or a negative control peptide (RLLFT-amide, Peptides International), was applied in parallel experiments as controls.

### Regulatory role of PAR1 in NSC differentiation *in vitro*

To determine the potential for PAR1 to regulate neural stem cell differentiation towards the oligodendrocyte lineage, we compared the expression of markers of each stage in neural stem cell cultures derived from PAR1+/+ or PAR1−/− mice using immunofluorescence staining approaches and quantitative real time RT-PCR.

#### Immunofluorescence for oligodendrocyte markers

Dissociated PAR1+/+ or PAR1−/− NSCs at passage 3 were plated as monolayers in triplicate on poly-L-lysine coated glass cover slips and grown for 2 DIV in media containing EGF and bFGF or for 5 DIV after growth factor withdrawal. At each end point, cultures were fixed with 2% paraformaldehyde and stained for NG2 (AB5320, EMD Millipore), Olig2 (AB9610, EMD Millipore), or PLP (ab28486, Abcam) using immunofluorescence techniques. Species appropriate fluorochrome conjugated secondary antibodies were obtained from Jackson ImmunoResearch (West Grove, PA). Sections were cover slipped with VECTASHIELD Hardset containing 4′,6-diamidino-2-phenylindole (DAPI, Vector Laboratories, Burlingame, CA). Five 20X microscopic fields encompassing the center and 4 poles of each coverslip were digitally imaged using an Olympus BX51 microscope (Olympus, Center Valley, PA). The mean number of NG2+ or Olig2+ cells was enumerated without knowledge of genotype and expressed as a ratio of the total number of DAPI+ cells counted in the same fields.

#### Real Time PCR for NSC differentiation markers

Dissociated PAR1+/+ or PAR1−/− NSCs at passage 3 were plated on poly-L-lysine coated 6 well plates for 2 DIV in the presence of EGF and bFGF, or for 3 or 7 DIV after withdrawal of growth factors to induce differentiation. The expression of RNA encoding Nestin for stem cell status, or Olig2, myelin basic protein (MBP), or proteolipid protein (PLP) for the oligodendrocyte lineage, were quantified using RT-PCR as described above. Primers specific for *Mus musculus* PLP forward, 5′-TCTTTGGCGACTACAAGACCAC-3′ and reverse, 5′-CACAAACTTGTCGGGATGTCCTA-3′, and for MBP forward, 5′-CCAGTAGTCCATTTCTTCAAGAACAT-3′ and reverse, 5′-GCCGATTTATAGTCGGAAGCTC-3′ were obtained from IDT. Primers for *Mus musculus* Olig2 (NM_016967; AB ID: Mm.PT.56a.42319010); GFAP (NM_010277.2) and Neurofilament 200 (NF(H), NM_010904.3) were from Applied Biosystems. To control for loading, all RNA expression levels were normalized to GAPDH amplified in the same samples as described above.

### Regulatory role of PAR1 on NSC proliferation and differentiation *in vivo*

To determine the impact of PAR1 on the proliferation and differentiation of NSCs *in vivo*, 8 wk old PAR1+/+ or PAR1−/− mice were perfusion fixed with 4% paraformaldehyde and the brains were embedded in paraffin. Six μm sections taken from a region +0.5 mm anterior to Bregma were deparaffinized and processed to localize Sox2, Ki-67 (550609, BD Bioscience), or Olig2 immunoreactivity within the SVZ using standard immunoperoxidase techniques. Numbers of Sox2−, Ki-67−, or Olig2-positive cells within the SVZ were counted bilaterally within the dorsolateral SVZ at the level where the lateral ventricle lies adjacent to the corpus callosum.

To determine whether changes in oligodendrocyte differentiation observed within the SVZ were reflected in white matter tracts of the adult brain, oligodendrocyte numbers were quantified in the corpus callosum and anterior commissure in the same or parallel tissue sections. Oligodendrocyte progenitors and young oligodendrocytes were identified by immunohistochemical localization of Olig2, and mature oligodendrocytes were identified as immunopositive for adenomatous polyposis coli (CC-1/APC1, AB16794, Abcam). GFAP-immunoreactive astrocytes (Z0334, Agilent Dako, Santa Clara, CA) were also enumerated in parallel sections. Cell counts were made bilaterally within the corpus callosum in the region immediately above the lateral ventricles. Counts within the anterior commissure were made in the region directly below each lateral ventricle. Antibody localization was visualized using standard immunoperoxidase techniques. All immunoperoxidase stained sections were counterstained with methyl green (Vector Laboratories) to visualize nuclei. Area measurements were made using Image J software^29^. Counts were made without knowledge of genotype and included at least 5 to 6 male mice in each case.

### Statistics

All statistical analysis was performed using SigmaPlot 13. An unpaired t-test (i.e. Student’s t-test) was performed to determine statistical significance. Statistical significance was defined as P < 0.05 and all data are represented as the mean ± SEM.

## Results

### Regulated expression of PAR1 by adult SVZ neural stem cells *in vivo* and *in vitro*

**Figure 1:**
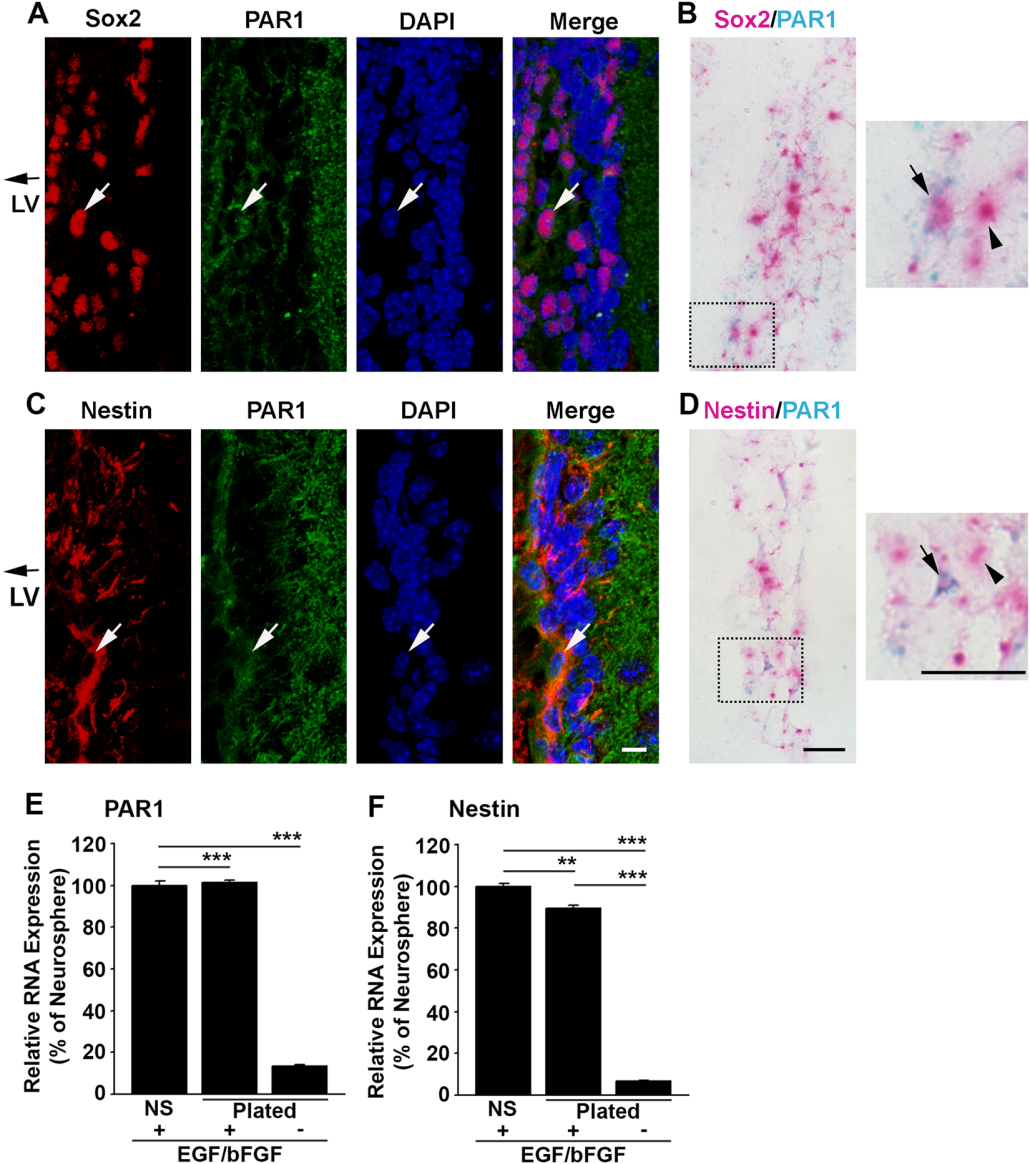
PAR1 is expressed by neural stem cells in the sub-ventricular zone (SVZ) of the adult mouse brain. Photomicrographs show immunofluorescence double-labeling for PAR1 with Sox2-positive (**A**), or PAR1 with Nestin-positive (**C**) multipotent neural stem cells (NSCs) within the lateral wall of the lateral ventricle (LV). RNAscope was used to identify cells expressing both PAR1 and Sox2 (**B**), or Nestin (**D**) RNA in NSCs of the adult SVZ. Arrow indicates an example of a double-labeled cell in each case, with arrowhead indicating a singly labeled cell (Scale bar = 10 μm). Boxed area in B and D is also shown at higher magnification to visualize double-labeled cells. (**E**) Histogram shows expression of PAR1 RNA was high in NSCs grown as neurospheres (NS), or when plated on poly-L-lysine coated coverslips as monolayers in stem cell media containing EGF and bFGF. PAR1 RNA expression by NSC monolayers decreased by 87% when EGF and bFGF were removed from the media for 7 DIV promoting stem cell differentiation. (**F**) Withdrawal of EGF and bFGF to induce NSC monolayer differentiation resulted in a parallel decrease in Nestin RNA expression. (**P < 0.01, ***P < 0.001 Students t-test).

To document PAR1 expression by NSCs of the adult mouse SVZ, immunofluorescence labeling was used to co-localize PAR1 to either Sox2- or Nestin-positive NSCs in the SVZ of the adult mouse brain (Fig. 1A,C). Sox2- or Nestin-immunofluorescence was observed in association with cells lining the wall of the lateral ventricles (LV) and a subset of these was also PAR1-immunopositive. Next, we confirmed the expression of PAR1 by SVZ NSCs using *in situ* hybridization to localize PAR1 RNA to NSCs expressing either Sox2 or Nestin RNA (Fig. 1B,D). Finally, we demonstrated that SVZ NSCs grown in culture as neurospheres or monolayers in the presence of EGF and bFGF express high levels of both PAR1 and Nestin RNA (Fig. 1E,F). Withdrawal of growth factors from monolayer cultures promoted differentiation and was paralleled by a 7.5-fold reduction in PAR1, and a 13-fold decrease in Nestin RNA expression (Fig. 1E,F, Students t-test P = 0.02 × 10^−4^). These findings collectively provide evidence that SVZ NSCs in the adult murine brain express PAR1 and that this receptor is substantially down regulated upon NSC differentiation.

### Thrombin gates Ca^2+^ signaling in SVZ neural stem cells by activation of PAR1

**Figure 2:**
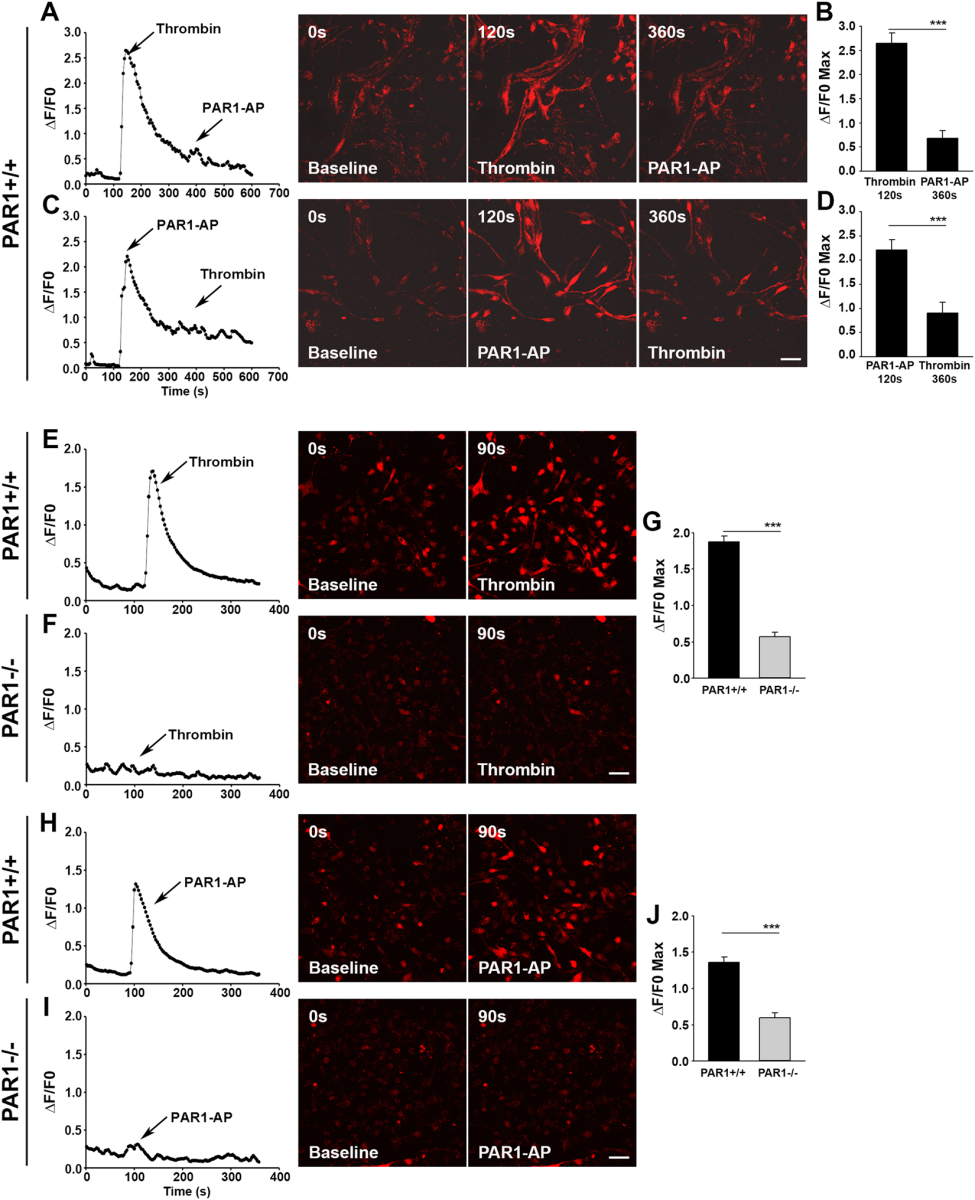
Thrombin gates Ca^2+^ signaling in neural stem cells in a PAR1-dependent manner. Traces, photomicrographs, and histograms (**A**–**J**), show that thrombin-induced Ca^2+^ signaling in NSCs occurs in a manner that depends in part on the presence of PAR1. The change in fluorescence intensity measured in response to application of (**A**) the PAR1 agonist thrombin (5 μg/mL, (135 nM)), or (**C**) a PAR1-activating peptide (PAR1-AP, 40 μM) to Rhod-3 loaded NSCs monolayer cultures grown in the presence of EGF and bFGF. Subsequent application of (**A**) PAR1-AP or (**C**) thrombin 240 s later resulted in significantly lower ΔF/F0, indicating receptor desensitization by the first agonist. Application of thrombin (**E**–**G**), or PAR1-AP (**H**–**J**), to NSCs derived from PAR1+/+ or PAR1−/− mice demonstrates the absence of Ca^2+^ signaling in response to either agonist in NSCs lacking PAR1. The change in intensity (ΔF) over baseline intensity (F0) is provided and expressed as ΔF/F0 = [(F − F0)/F0]. (***P < 0.001, Scale bar = 50 μm).

The ability of neural stem cell PAR1 to gate Ca^2+^ signaling was evaluated by determining thrombin or PAR1-AP elicited changes in fluorescence intensity in Rhod-3 loaded NSCs (Fig. 2A–J). In addition, receptor cross desensitization was performed to determine the extent to which each agonist gated Ca^2+^ specifically through activation of PAR1. Thrombin (135 nM) elicited a sharp increase in fluorescence intensity over that seen at baseline, and application of PAR1-AP 240 s after the application of thrombin resulted in a 3.8-fold lower Ca^2+^ response (Fig. 2A,B, P = 0.02 × 10^−7^, Students t-test). Similarly, application of PAR1-AP (40 μM) elicited a robust Ca^2+^ response, and a 2.4-fold reduction in Ca^2+^ signaling was observed when thrombin was applied 240 s after PAR1-AP (Fig. 2C,D, P = 0.0002, Students t-test). Reductions in fluorescence intensity observed when an agonist is applied after the first agonist has already activated PAR1, indicate receptor desensitization and point to the ability of the first agonist to specifically activate PAR1.

To confirm PAR1-gated Ca^2+^ signaling, NSCs derived from PAR1 deficient mice were examined. Rhod-3 loaded PAR1+/+ or PAR1−/− NSCs were imaged for 1.5 min to establish the baseline fluorescence prior to application of either thrombin (135 nM), or PAR1-AP (40 μM), with changes in fluorescence monitored over the subsequent 3.5 min. Increases in fluorescence intensity elicited by each agonist were observed in PAR1+/+ NSCs. In contrast, thrombin or PAR1-AP did not elicit Ca^2+^ signaling in PAR1−/− NSCs (Fig. 2E–J, P = 0.05 × 10^−8^). These results suggest that functional PAR1 is expressed by SVZ neural stem cells derived from the adult brain, and that both thrombin and PAR1-AP specifically activate PAR1 in NSCs to gate Ca^2+^ signaling.

### Inhibition of PAR1 increases neural stem cells proliferation

**Figure 3:**
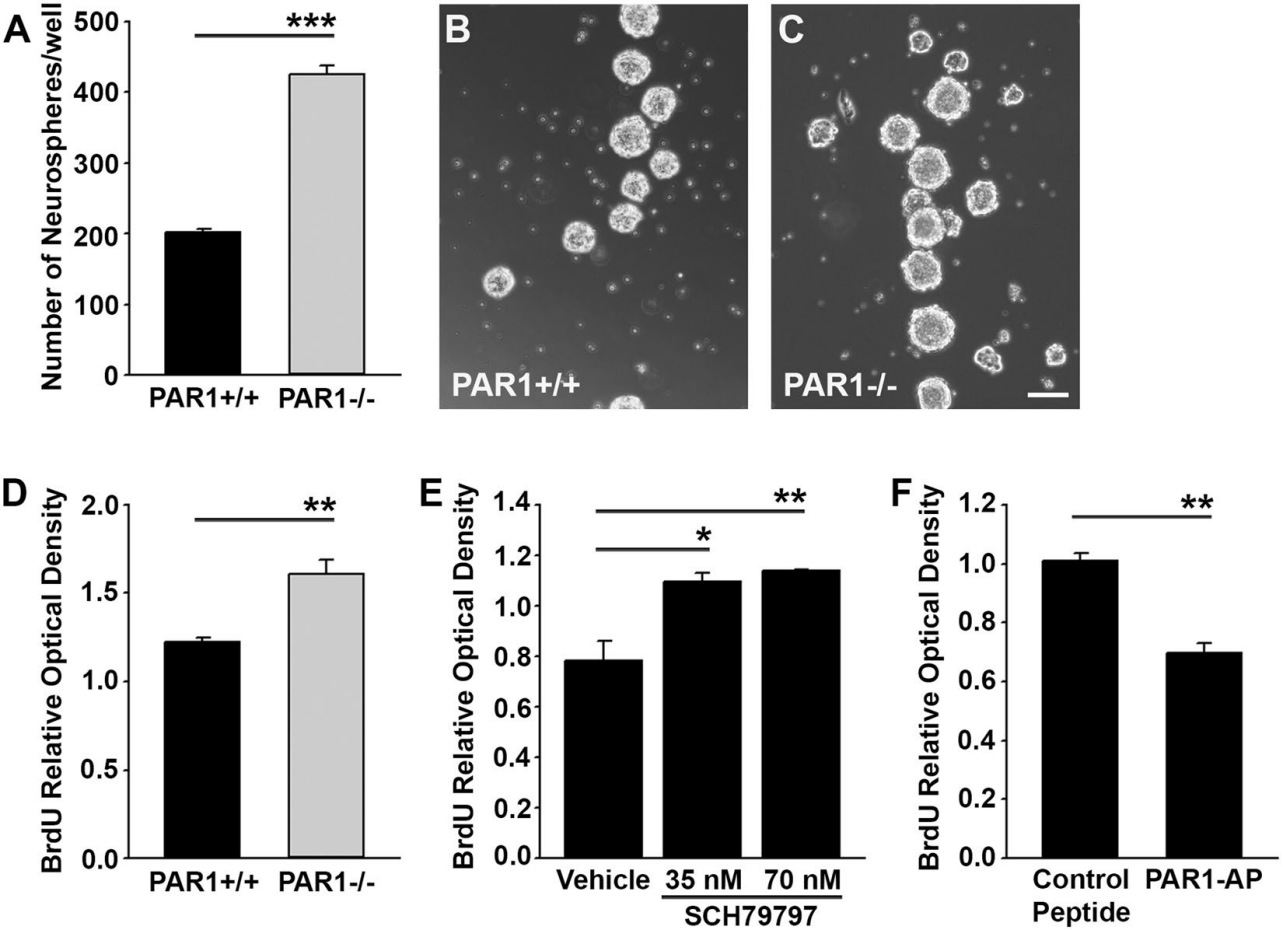
Blocking PAR1 promotes neural stem cell proliferation. Histograms and photomicrographs (**A**–**D**) show that NSCs lacking PAR1 have increased capacity to form neurosphere (NS) in culture and demonstrate higher levels of BrdU incorporation compared to those derived from PAR1+/+ mice (Scale bar = 100 μm). Treatment of NSCs with a PAR1 small molecule inhibitor, SCH79797 (35 or 70 nM) for 18 h increased BrdU incorporation (**E**), whereas a PAR1 activating peptide (PAR1-AP, 40 μM) reduced BrdU incorporation (**F**). (*P < 0.05, **P < 0.01, ***P < 0.001 Students t-test).

The potential activity of SVZ adult neural stem cell PAR1 in regulating proliferation was evaluated by comparing the impact of PAR1 genetic deletion on neurosphere formation and BrdU incorporation *in vitro* (Fig. 3A–D). The plating of 5000 PAR1+/+ neural stem cells in EGF and bFGF containing media for 7 d resulted in 201 ± 5.2 spheres, compared to 425 ± 12.1 in the case of PAR1−/− NSCs, a 2.1-fold increase (Fig. 3A–C, P = 7.0 × 10^−4^, Students t-test). PAR1−/− NSCs grown in growth factor containing media also incorporated 30% more BrdU compared to PAR1+/+ cells (Fig. 3D, P = 0.009, Students t-test). We next tested whether a PAR1 small inhibitor would also promote expansion of SVZ NSCs *in vitro*. SCH79797 (35 or 70 nM), a non-peptide PAR1 specific antagonist, promoted a 39% increase in BrdU incorporation in PAR1+/+ cells (Fig. 3E, P ≤ 0.02, Students t-test). Conversely, activation of PAR1 with PAR1-AP (40 μM) reduced incorporation of BrdU by 30% (Fig. 3F, P = 0.007, Students t-test). Together these findings suggest that PAR1 expression by neural stem cells suppresses their proliferative capacity.

### PAR1 knockout enhances NSC production and glial differentiation *in vitro*

**Figure 4:**
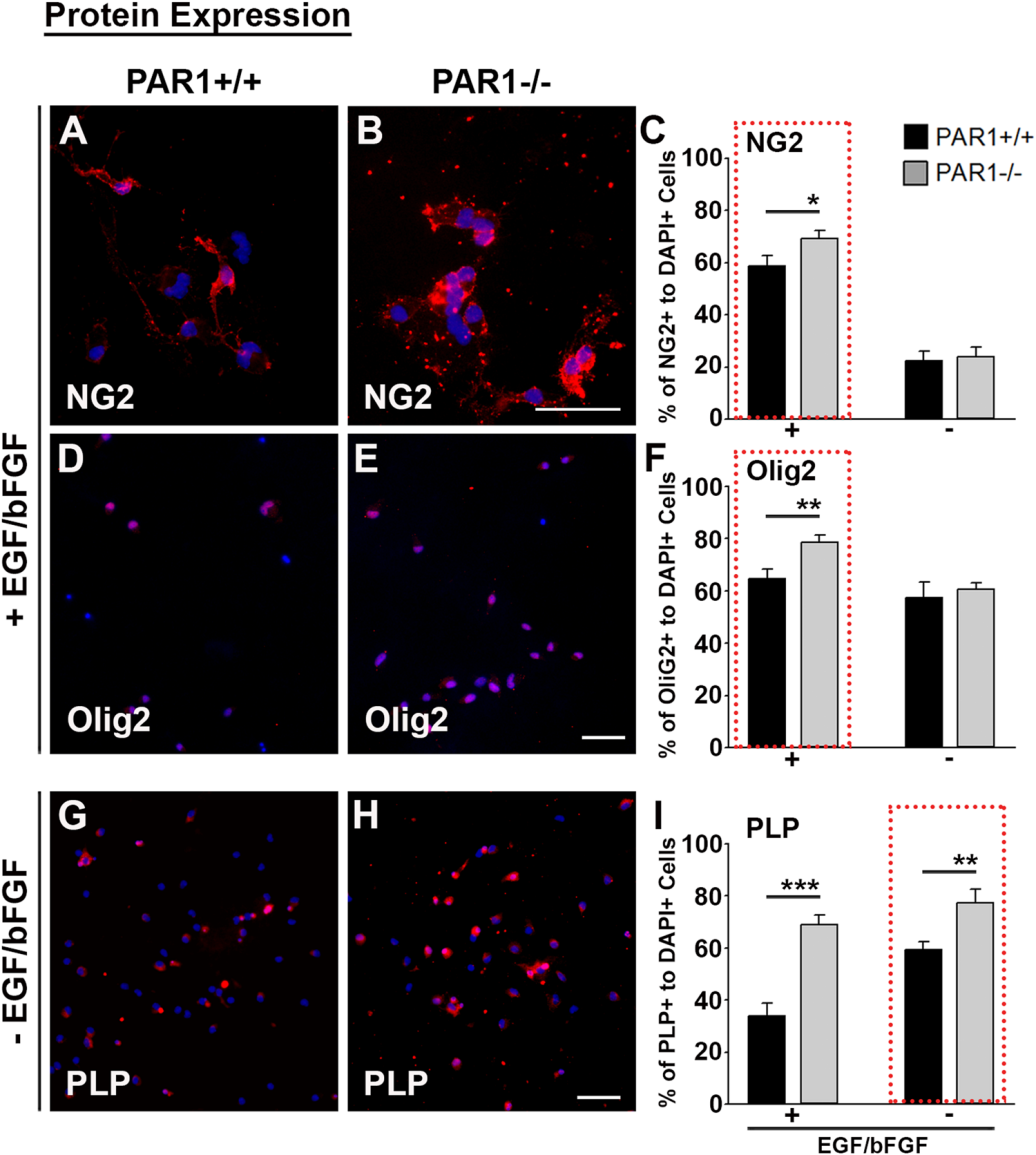
PAR1 gene knockout enhances neural stem cell expansion and oligodendrocyte differentiation in cell culture. Photomicrographs and cell counts normalized to DAPI show significantly increased immunofluorescence for NG2, a marker for NSCs (**A**–**C**), Olig2 a marker of oligodendrocyte progenitor cells and young oligodendrocytes (**D**–**F**), and PLP, a marker for mature oligodendrocytes (**G**–**I**) in NSC cultures grown in the presence or absence of EGF and bFGF. The percentage of NG2, Olig2 and PLP+ cells was greater in NSC cultures lacking PAR1 when grown in the presence of EGF and bFGF (2 DIV). After withdrawal of EGF and bFGF for 5 DIV, we observed an expected decrease in the percentage of cells positive for NG2 and an increase in those positive for PLP. The increase in PLP+ cells after withdrawal of EGF and bFGF was greater in NSCs lacking PAR1 compared to wild type NSCs. (Scale bar = 50 μm). (*P < 0.05, **P < 0.01, ***P < 0.001, Students t-test).

**Figure 5:**
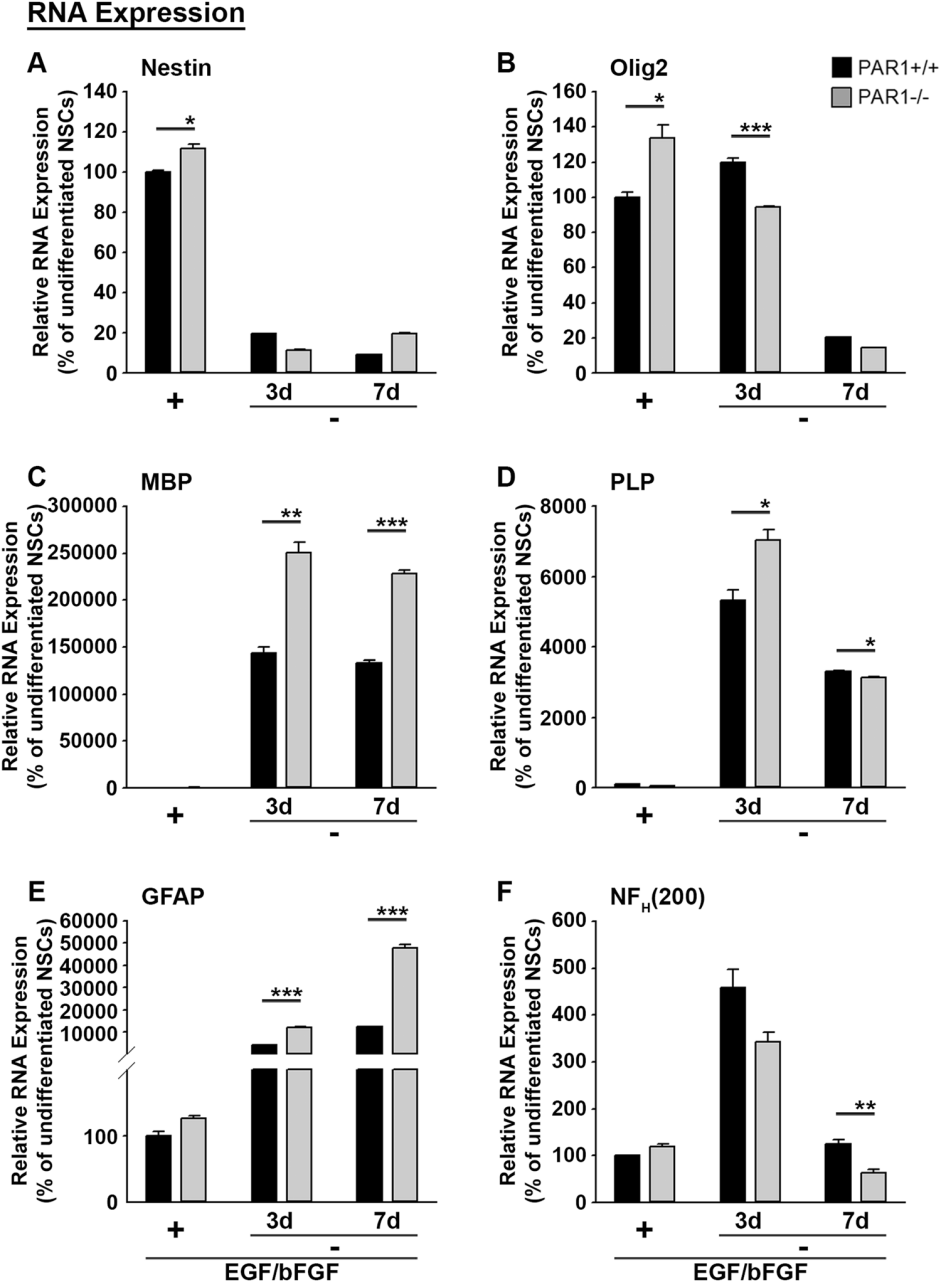
SVZ NSCs with PAR1 gene knockout show enhanced differentiation *in vitro*. (**A**) Expression of RNA indicative of the neural stem cell stage of differentiation (Nestin) was greater in NSCs derived from the SVZ of adult PAR1−/− compared to PAR1+/+ mice when grown in the presence of EGF and bFGF. The rapid loss of Nestin RNA in NSCs by 3 and at 7 d after withdrawal of EGF and bFGF is consistent with their differentiation and did not differ across genotypes. (**B**) Olig2, a marker of oligodendrocyte progenitor cells and young oligodendrocytes was higher in PAR1−/− NSCs grown in the presence of EGF and bFGF and lower by 3 d after growth factor withdrawal. Expression of RNA encoding MBP (**C**) and PLP (**D**) were greatly increased by growth factor withdrawal and each was expressed at highest levels in NSCs lacking PAR1 at 3 d, with MBP remaining significantly higher after 7 d of differentiation. (**E**) Growth factor withdrawal also resulted in an increase in GFAP (**E**) and Neurofilament (**F**) expression, with differentiating NSCs lacking PAR1 showing higher levels of GFAP but lower levels of neurofilament. (*P < 0.05, **P < 0.01, ***P < 0.001 Students t-test).

Previously, we reported that mice with PAR1 gene knockout show enhancements in spinal cord myelination, including an accelerated pattern of PLP production developmentally and higher levels of MBP in adulthood^16^. To determine the potential influence of PAR1 on the production of oligodendrocyte lineage cells from NSCs, SVZ NSCs were plated as monolayers on poly-L-lysine coated glass coverslips in stem cell media containing EGF and bFGF, or under conditions where EGF and bFGF were withdrawn to elicit differentiation (Figs 4 and 5). Markers of oligodendrocyte differentiation were quantified 5 d later using immunofluorescence. First, SVZ NSC monolayers derived from PAR1−/− mice showed a 1.2-fold increase in the number of cells stained for markers of OPCs (NG2 and Olig2) (P < 0.03, Students t-test), an effect that was lost with growth factor withdrawal and differentiation. Interestingly, a greater number of cells in NSC monolayers derived from PAR1−/− mice were also stained for the major myelin protein PLP, both in the presence of growth factors (2-fold) and after their withdrawal (1.3-fold higher compared to PAR1+/+) (P ≤ 0.006, Students t-test).

Next we used quantitative real time PCR to determine the impact of PAR1 gene knockout on the expression of neural stem cell, oligodendrocyte, astrocyte and neuron-specific markers in NSC monolayers grown in 6 well plates in the presence or absence of EGF and bFGF (Fig. 5). Nestin RNA expression was 1.1-fold higher in PAR1−/− compared to PAR1+/+ cells when grown in stem cell media with EGF and bFGF (Fig. 5A, P = 0.01 Students t-test). Nestin RNA expression was extinguished upon differentiation by 3 and after 7 d, in both PAR1+/+ and PAR1−/− NSC monolayers. Olig2 RNA levels were 1.3-fold higher in PAR1−/− SVZ monolayers grown in the presence of EGF and bFGF (P = 0.01), but were 1.3-fold lower than PAR1+/+ after 3 d of differentiation (Fig. 5B, P = 0.0007). The expression of MBP and PLP RNA by NSC monolayers was highly increased at 3 and 7 d after withdrawal of growth factors. NSC monolayers lacking PAR1 showed 1.3-fold higher levels of PLP at 3d, and 1.7-fold higher levels of MBP at 3 and 7 d of differentiation, compared to PAR1+/+ NSC monolayers (Fig. 5C,D, P ≤ 0.001, Students t-test). GFAP RNA expression levels were 3 to 4-fold higher in PAR1−/− NSCs at 3 and 7 days of differentiation.

By contrast, the abundance of Neurofilament RNA was 2-fold lower in cultures of PAR1−/− NSCs 7 d after growth factor withdrawal (P = 0.006, Students t-test). Together these results suggest that knocking out PAR1 in SVZ NSC cultures can increase the expression of markers of mature oligodendrocytes and astrocytes upon differentiation.

### Increases in NSCs and oligodendrocyte progenitors occur in the SVZ of adult mice with PAR1 loss-of-function

**Figure 6:**
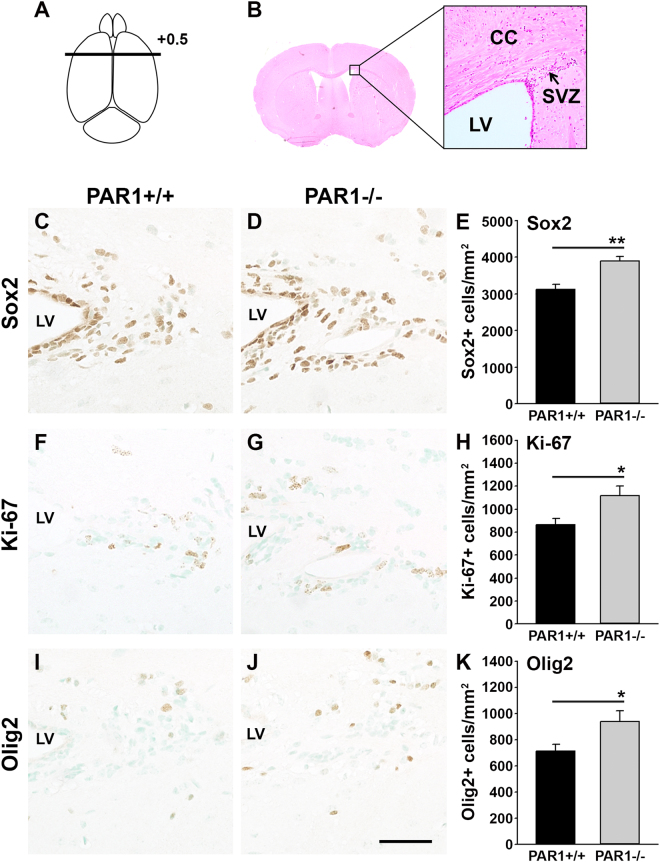
PAR1 gene knockout results in increased numbers of Sox2+ neural stem cells and oligodendrocyte progenitor cells in the adult SVZ. Counts of Sox2-positive NSCs (**C**–**E**), and Ki-67-positive proliferating cells (**F**–**H**) were greater in coronal sections taken +0.5 mm to Bregma (**A**,**B**) through the SVZ of PAR1−/− compared to PAR1+/+ adult mice. A greater number of cells positive for the early oligodendrocyte lineage marker Olig2 were also observed in PAR1−/− mice in parallel sections (**I**–**K**). Ki-67 and Olig2 stained sections were counterstained with methyl green. (*P < 0.05, **P < 0.01, Students t-test) (Scale bar = 50 μm).

To determine if the regulatory actions of PAR1 on NSC expansion and differentiation observed in cell culture also occur *in vivo*, we quantified neural stem cells and Olig2+ oligodendrocyte progenitors in the SVZ of adult PAR1+/+ and PAR1−/− mice using immunohistochemical approaches (Fig. 6). The SVZ of PAR1 knockout mice contained 1.2-fold more Sox2+ neural stem cells compared to wild type mice (Fig. 6C–E, P = 0.002, Student’s t-test). This was accompanied by a 1.3-fold increase in counts of Ki-67+ cells in the SVZ of PAR1−/− mice compared to PAR1+/+ in near adjacent sections (Fig. 6F–H, P = 0.03, Student’s t-test). The SVZ of mice lacking PAR1 also showed a 1.3-fold increase in the number of cells immunopositive for Olig2 (Fig. 6I–K, P = 0.04, Student’s t-test).

### Increases in oligodendrocyte progenitors and mature oligodendrocytes are found in white matter tracts of adult mice lacking PAR1

**Figure 7:**
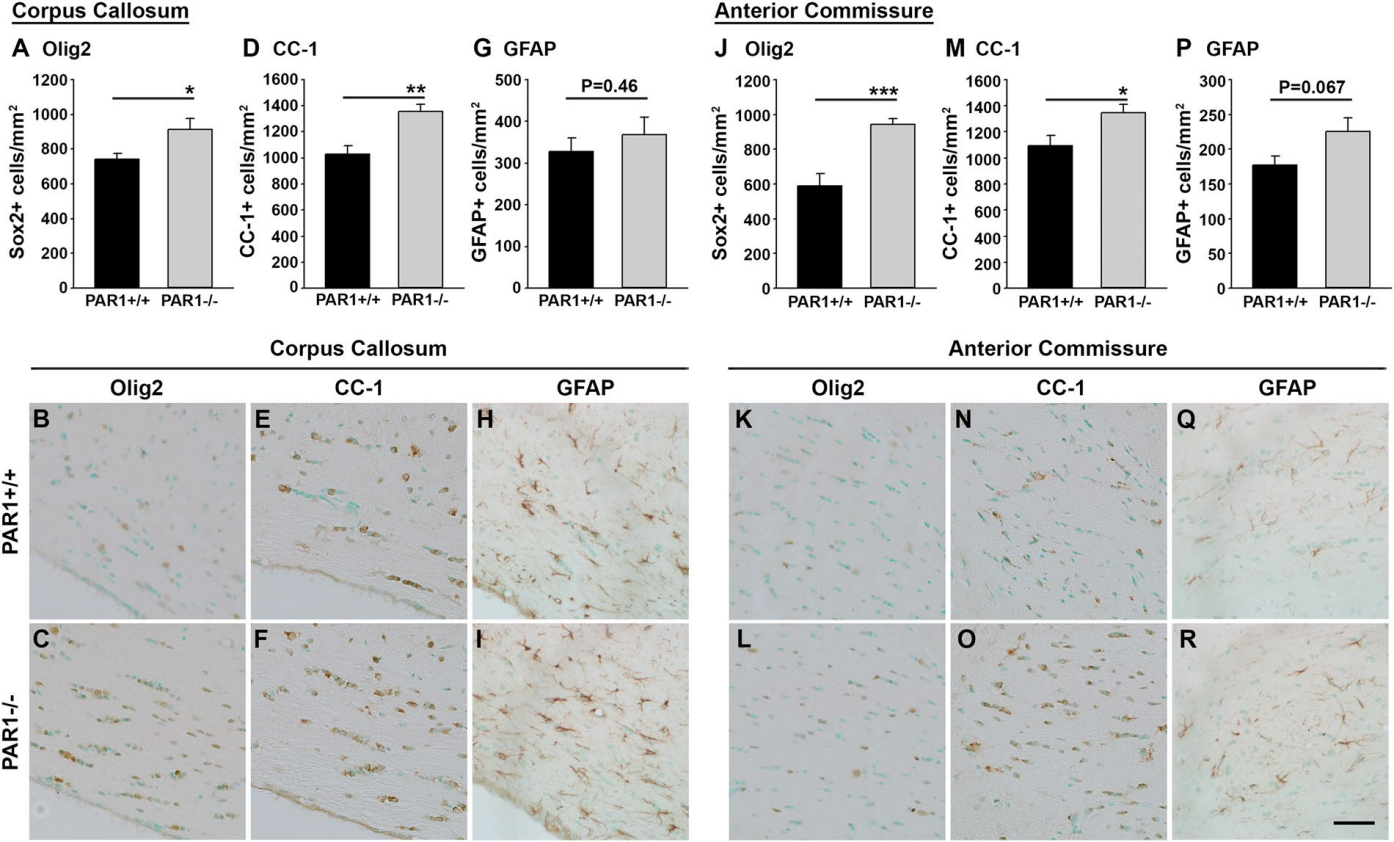
PAR1 gene knockout results in increased numbers of oligodendrocytes in the corpus callosum and anterior commissure of the adult mouse brain. The corpus callosum of adult (8 wk) PAR1−/− mice showed an increase in the number of Olig2- (**A**–**C**) and CC-1-positive cells (**D**–**F**) relative to PAR1+/+ mice. Similar increases in Olig2 (**J**–**L**) and CC-1 (**M**–**O**) were also observed in the anterior commissure of mice with PAR1 gene knockout. Counts of GFAP+ astrocytes in parallel sections did not differ between genotypes (**G**–**I** and **P**–**R**). All data are expressed as the mean number of positive cells counted per area ± SEM. (*P < 0.05, **P < 0.01, ***P < 0.001, Students t-test) (Scale bar = 50 μm).

To establish if the increased numbers of NSCs and OPCs observed in the SVZ of PAR1−/− mice may contribute to increases in myelinating cells in white matter tracts of the adult brain, we quantified cells immunoreactive for Olig2, a marker of OPCs and young oligodendrocytes and CC-1, a marker of mature myelinating cells, in the corpus callosum and the anterior commissure. We also enumerated the number of GFAP+ astrocytes in the same regions. The number of Olig2-immunoreactive cells in the corpus callosum was increased by 1.2-fold in PAR1−/− compared to PAR1+/+ mice (Fig. 7A–C, P = 0.03, Student’s t-test). In addition, 1.3-fold more CC-1-immunoreactive mature oligodendrocytes were observed in the corpus callosum of adult mice lacking PAR1 (Fig. 7D–F, P = 0.002, Student’s t-test). Similar increases in both Olig2 (Fig. 7J–L, 1.6-fold, P = 0.0009, Student’s t-test) and CC-1 (Fig. 7M–O, 1.2-fold, P = 0.02, Student’s t-test) were observed in the anterior commissure of PAR1−/− compared to PAR1+/+ mice. By contrast, counts of GFAP-immunoreactive astrocytes in the same regions were not significantly different between genotypes (Fig. 7G–I and P–R). These findings suggest that the increases in myelinating cells we previously reported in the spinal cord of mice lacking PAR1^16^ extend to white matter tracts of the adult brain. Also, taken with findings suggesting that blocking SVZ PAR1 can enhance the production of myelinating progenitor cells, the increases in oligodendrocyte progenitors and myelinating cells in white matter tracts of the adult brain may manifest at least in part from neural stem cell sources.

## Discussion

Stem cells derived from the SVZ of the adult brain play key roles in the replacement of post mitotic cells, including a prominent role in the generation of new OPCs that form the substrate for myelin regeneration. While there has been considerable research regarding the SVZ as a source of OPCs and their migration, much less is known regarding the microenvironment of the stem cell niche^30^. Here we investigate how PAR1, the high affinity receptor for thrombin, regulates SVZ NSC expansion and differentiation with a focus on the oligodendrocyte lineage. PAR1 is a GPCR that upon cleavage is able to translate changes in the proteolytic microenvironment into adaptive or in some cases maladaptive cellular responses. Our findings suggest that PAR1 activation plays a suppressive role in the expansion of NSCs derived from the SVZ of the adult mouse brain and their capacity to differentiate towards mature myelinating oligodendrocytes *in vivo*. First we document functional expression of PAR1 by SVZ NSCs. We observe that the SVZ of PAR1 knockout mice contains greater numbers of both NSCs and OPCs and increased numbers of OPCs and mature oligodendrocytes are seen in white matter tracts of the adult brain. Accordingly, genetic or pharmacologic PAR1-loss-of-function in SVZ NSCs *in vitro* promotes proliferation and increases in the abundance of mature myelinating oligodendrocytes *in vivo*. The discovery of an essential role for PAR1 in NSC production and differentiation identify this highly druggable receptor as an important biological node for manipulation to enhance endogenous expansion of NSCs in the SVZ and their myelinating potential, to protect NSCs from the actions of thrombin, and to potentially to foster the success of neural stem cell transplantation strategies.

### Regulated expression of functional PAR1 by SVZ neural stem cells

Consistent with a role for PAR1 as a regulator of SVZ NSC physiology, we document PAR1 expression by both Sox2+ and Nestin+ NSCs in the SVZ of the adult forebrain *in vivo*. SVZ neurosphere or monolayer cultures also express high levels of PAR1 in the presence of EGF and bFGF. The dramatic reductions in PAR1 expression by NSCs upon growth factor withdrawal and differentiation *in vitro* is consistent with a model in which the intact receptor plays roles at progenitor stages with receptor loss-of-function positioned to influence differentiation. The reductions in PAR1 as differentiation proceeds mirror PAR1 expression dynamics we observe in other developing systems. For example, OPCs isolated from the postnatal mouse cortex express high levels of PAR1 with dramatic reductions occurring upon differentiation *in vitro*^16^. Correspondingly, the spinal cord of mice contains very little myelin and high levels of PAR1 expression at birth, with receptor levels progressively decreased as myelination ensues over the first 3 postnatal weeks^16^.

To confirm that PAR1 expressed by SVZ NSCs is functional, we determined the ability of thrombin or a PAR1-specific activating peptide (PAR1-AP), to gate Ca^2+^ signaling in wild type or PAR1 knockout NSC monolayers. These experiments demonstrate that PAR1 is necessary and sufficient to gate Ca^2+^ downstream of thrombin or PAR1-AP in NSCs derived from the adult SVZ. Although thrombin is able to activate PAR3 and PAR4, and even PAR2 at higher concentrations^8^, in NSC monolayers grown in the presence of EGF and bFGF, genetic knockout of PAR1 completely blocked the ability of thrombin to gate intracellular Ca^2+^ flux. As Ca^2+^ ions are ubiquitous second messengers with wide-ranging roles in signal transduction, future studies are needed to delineate how PAR1-mediated increases in intracellular Ca^2+^ modulate the proliferative and differentiation effects we observe in SVZ-derived NSCs.

### PAR1 loss-of-function promotes neural stem cell expansion

NSCs from the adult SVZ can be grown in suspension in the presence of EGF and bFGF to promote self-renewal and to maintain an undifferentiated multipotent state^31–33^. We report here for the first time that genetic knockout of PAR1 results in a doubling of neurosphere numbers in culture and this is reflected in an increase in BrdU incorporation. We additionally demonstrate that application of a PAR1 small molecule antagonist to wild type NSCs replicates the effects of PAR1 knockout, resulting in a significant enhancement of BrdU incorporation in neurosphere cultures. The presence of PAR1 is necessary and sufficient to impede proliferation of neurospheres in culture since PAR1-AP that mimics the tethered ligand of the receptor attenuates SVZ NSC proliferation. Near parallel effects were recently documented in cultures of neural precursor cells from the murine hippocampal dentate gyrus, with thrombin or PAR1-AP reducing proliferation^17^. Collectively, these studies suggest that PAR1 activation is necessary and sufficient to suppress NSC proliferation *in vitro*. To determine whether the regulatory effects of targeting PAR1 on NSC expansion *in vitro* extend to the adult murine brain, we made counts of Sox2+ NSCs in the SVZ of wild type and PAR1 KO mice. Consistent with a role for PAR1 in suppressing NSC expansion *in vivo*, higher numbers of Sox2+ and Ki-67+ cells are present in SVZ of adult PAR1 knockout mice.

### PAR1 loss-of-function increases the abundance of mature oligodendrocytes *in vitro* and *in vivo*

In the adult brain mature oligodendrocytes can be replaced from two primary reservoirs. First, myelinating cells can be replaced from a pool of OPCs that reside in a widely distributed manner within the parenchyma of the CNS and that are estimated to comprise 5-6% of all neural cell types^34,35^. Secondly, neural precursor cells located primarily in the dorsal and lateral walls of the SVZ may give rise to new pre-myelinating OPCs^36–38^. Despite the presence of these myelinating cell reservoirs, multiple studies indicate that OPCs may already be present at sites of demyelination, but in many cases, especially chronic MS lesions, these fail to differentiate. For example, although endogenous remyelination occurs at early stages in MS^39–41^, it increasingly fails with disease progression^42–48^. Understanding factors that regulate OPC differentiation is highly significant since although many years of translational research have generated immunomodulatory drugs to treat MS, there are still no clinically approved treatment strategies to promote myelin repair.

To determine if PAR1 plays a regulatory role in the production of mature oligodendrocytes from NSCs, we quantified stem cell and myelinating cell markers in NSC cultures grown as monolayers in the presence or absence of EGF and bFGF. After growth factor withdrawal to induce differentiation, the expression of the two major myelin proteins, MBP and PLP were each nearly two-fold higher in PAR1 knockout NSC cultures. Moreover, enhancements in markers of oligodendrocyte differentiation in PAR1 knockout NSCs were observed even in the presence of EGF and bFGF, including Olig2 and PLP. Reductions in NSC PAR1 expression with growth factor withdrawal *in vitro* and as CNS myelination unfolds^16^, lend support to the concept that PAR1-loss-of function is associated with oligodendrocyte differentiation. Consistent with this, we observe higher numbers of Olig2 and CC-1+ oligodendrocytes in the corpus callosum and anterior commissure of mice lacking PAR1. These new findings suggest that the enhancements we recently reported in myelin production in the spinal cord of mice lacking PAR1^16^ are generalizable to broad regions of the CNS. Future studies are needed to clearly distinguish the extent to which the increases in the abundance of myelin producing cells we observe *in vivo* and *in vitro* in the absence of PAR1 are related to enhancements of NSC proliferation and to extent to which they also include a direct impact on NSC differentiation.

Findings that PAR1 loss-of-function enhances the abundance of myelin producing cells in cultures of differentiating SVZ NSCs do not exclude potential effects on other lineages. To begin to address this in the current study, we examined levels of GFAP expression in differentiating wild type and PAR1 knockout NSCs in cell culture and made counts of GFAP+ astrocytes in white matter tracts of the adult brain across genotypes. While counts of GFAP-immunoreactive cells in the corpus callosum and anterior commissure did not differ between wild type and PAR1 knockout mice, NSC cultures with PAR1 loss-of-function showed increased levels of GFAP expression upon growth factor withdrawal. By contrast neurofilament, a marker for neurons, was decreased in PAR1−/− NSC cultures upon differentiation. These results suggest that under the culture conditions of this study that the effects of PAR1 knockout extend beyond myelinating cells to include astrocytes, but additional studies using a neural cell lineage-specific culture conditions will be needed to confirm these *in vitro* findings and extend them to other cell types. While we observed enhancements in oligodendrocyte lineage cells, but not GFAP+ astrocytes in the corpus callosum and anterior commissure of PAR1−/− mice, future studies are needed to examine a greater number of brain regions and cell types, and to apply fate-mapping approaches to capture the full spectrum of regulatory actions of PAR1 in NSC differentiation in the adult brain.

### Clinical significance

Findings presented suggesting that PAR1 activation can block SVZ NSC expansion and differentiation have important implications for neuropathological conditions in which PAR1 activators become elevated in the CNS. As the high affinity receptor for thrombin it is not difficult to envision how PAR1 may be aberrantly activated with changes in blood brain barrier permeability and serum protein extravasation. PAR1 may also be activated by other proteinases at lower affinity, including MMP1, plasmin and kallikrein 6. Like thrombin, other PAR1 activators are present in the general circulation and/or enriched in CSF and may also be dynamically up regulated within the parenchyma of the CNS itself in the context of CNS injury and disease^49–51^. For example, kallikrein 6 is highly enriched in the serum^52^ and CSF of MS patients^53–55^. Findings presented here highlight the potential consequences of aberrant PAR1 activation for expansion of NSCs in the SVZ niche and their capacity to form mature myelinating cells. The results of this study also suggest that clinical strategies aimed at replacement of myelin by targeting endogenous myelinating cell reservoirs, or through transplantation strategies^56,57^, may be enhanced by additionally targeting PAR1. These studies also set the stage for examination of the effects of PAR1 on the differentiation of other cell types.

As is the case for OPCs, evidence suggests that oligodendrogenesis from NSCs is regulated by both positive and negative regulators. Ciliary neurotrophic factor^58^, EGF^59,60^, FGF^61^ and Wnt^62,63^ all increase oligodendrogenesis from SVZ NSCs, while BMPs^64^ are inhibitory. Targeting NSC transcription factors can also exert oligodendrogenic effects with over expression of Olig2^65–67^, Nkx2.2^68^ and Sox10^67^ promoting enhancements, but Sirtuin 1^69^, Gli1^70^ and neurofibromin 1^71,72^ being inhibitory. Given that PAR1 activation elicits a conformational change in the receptors intracellular loops that results in specific interactions with Gα subunits G12/13, Gq/11 or Gi, or Gβγ, resulting in modulation of signaling through Rho-guanine nucleotide exchange factors, phosphoinositide phospholipase C, mitogen-activated protein kinase, adenylate cyclase, or Phosphatidylinositol-4,5-bisphosphate 3-kinase pathways^73^ an important line of future studies will be to determine to what extent PAR1 signaling integrates with signaling elicited by growth factors already know to play key roles in stem cell and oligodendrocyte expansion and differentiation.

A limitation of this study is that we cannot definitively state whether the effects we observe on NSC expansion and OPC differentiation as a result of PAR1 knockout are related to changes in PAR1 on NSCs alone or if other cell types are involved. The neurogenic stem cell niche of the CNS includes neural stem/progenitor cells, blood vessel cells, ependymal cells, astrocytes, microglia and oligodendrocytes. Specific cell interactions involving PAR1 are likely to be important. For example, PAR1 is densely expressed by endothelial cells and these cells have been shown to influence NSC self-renewal and migration through the action of a yet to be identified secreted diffusible factor^74,75^. Deciphering the role(s) of PAR1 both intrinsic and extrinsic to NSCs awaits future studies using conditional gene targeting approaches.

## Conclusion

Taken with our prior studies documenting the pro-myelinating effects of targeting PAR1 during spinal cord development, the current findings pointing to parallel effects in the adult brain that include a direct impact on SVZ neural stem cells underscore the fundamental importance of PAR1 in myelination across the lifespan and across brain regions. These data provide a working hypothesis for the role of PAR1 in myelin production in the CNS that yields a number of testable predictions. In particular, experiments to determine whether PAR1 can be targeted to foster myelin regeneration after injury are now needed to determine how these findings can be translated to promote recovery of function. In addition, future lineage-tracing studies will be important to establish whether blocking PAR1 function also affects the production and/or differentiation of other glial and neuron cell types that derive from the SVZ of the adult brain. Moreover, by identifying PAR1 as an essential regulator of neural stem cell expansion in the adult brain these studies have wide ranging implications for use of PAR1 targeting strategies to enhance neural stem cell based regeneration strategies for endogenous precursors or transplanted cells.
